# “We literally worked in parking lots, cars, garages, and separately set up party tents”: qualitative study on the experiences of GPs in the frame of the SARS-CoV-2 pandemic in Austria

**DOI:** 10.1186/s12913-023-10363-4

**Published:** 2023-12-12

**Authors:** Susanne Rabady, Mira Mayrhofer, Nathalie Szabo, Patrick Erber, Kathryn Hoffmann

**Affiliations:** 1 Division General and Family Medicine, Department of General Health Studies, Karl Landsteiner University of Health Sciences, Dr. Karl-Dorrek-Straße 30, Krems, 3500 Austria; 2 Department of Primary Care Medicine, Center for Public Health, Medical University of Vienna, Vienna, Austria

**Keywords:** Pandemic, COVID 19, Primary health care, General practitioner, Pandemic management

## Abstract

**Background:**

Primary care is internationally recognised as one of the cornerstones of health care. During the COVID-19 pandemic, primary care physicians were assigned a variety of tasks and thus made a significant contribution to a country’s pandemic response. They were expected to perform a variety of tasks, such as diagnosing and treating people with COVID-19, maintaining health care for all other patients, as well as several public health tasks, such as diagnostic testing and vaccination, protecting patients and staff from infection, and serving as community trusted persons. In Austria, there are no structured levels of care, no definition of the role of the general practitioner during a pandemic is given, and no specific support structures are present. The aim of this study was to assess the views and experiences of primary care physicians regarding supportive and hindering factors for pandemic preparedness in Austria.

**Methods:**

Qualitative study using semi-structured interviews. A total of 30 general practitioners were interviewed, with particular attention to an equitable distribution in small, medium and large primary care facilities. Qualitative content analysis was performed.

**Results:**

Interviewees described a wide range of infection control, organisational and communication measures that they had implemented. They made changes to practise equipment, found makeshift solutions when supplies were scarce, and established communication and information pathways when official communication lines were inadequate.

**Conclusion:**

General practitioners took on essential tasks and showed a high level of understanding of their role in the pandemic response. This was achieved mainly at an informal level and with high personal commitment. Their functioning in the absence of structural regulations and support shows that they had a clear intrinsic understanding of their responsibilities. To ensure reliability and sustainability and to reduce their burden, it will be necessary to clarify the role and tasks of a general practitioner and to provide the necessary support. This concerns both infrastructural support and communication and information strategies. As part of the reform to strengthen primary care, primary care needs to be seen, valued and involved in decision-making processes.

**Supplementary Information:**

The online version contains supplementary material available at 10.1186/s12913-023-10363-4.

## Background

Even before the COVID-19 pandemic, it was clear that a well-developed primary care sector, as defined by the Expert Panel on Effective Ways of Investing in Health [1], is essential for comprehensive, cost-effective and equitable health for any population [1–4]. The Austrian health care system is characterised by free access to almost all levels of health care and a lack of regulation of the responsibilities of these levels, including primary care, which is mostly informal and thus left to self-organisation. This also means that there is no clearly defined role for primary health care (PHC) in pandemic management, no integration into official communication channels within the health system, and no distribution plans for personal protective equipment (PPE) [5]. Even a first review of Austrian pandemic management, published in 2021, paid very little attention to the primary care (PC) sector, devoting half a page out of 150 to it. This was at a time when the WHO had already specified the role of PC in COVID-19 management [6].

Several studies have looked at the impact of the pandemic and its management on primary care in different countries [7–10], such as Germany and the Scandinavian countries. Some studies compared different countries with different health care systems [11–13]. A study of Austria’s performance at primary care level has been lacking until now.

The COVID-19 pandemic demonstrated that a strong primary care sector, including general practice, is one of the most important pillars of pandemic control. The core functions of PHC as outlined by WHO (Interim Guidance) are: Timely, effective and safe supportive management of patients with suspected and confirmed COVID-19 at the primary care level; provision of essential health services at the primary care level and COVID-19 vaccination.

Specifically, the following tasks are assigned to primary care structures and are considered essential for pandemic management by international studies and analyses: reducing the burden on other levels of health care, e.g. hospitals and nursing homes [3], avoiding unnecessary consultations at different points of care reduces the risk of infection for the individual: testing, isolation and treatment of patients can be completed in a single setting [6, 11, 14]. Good surveillance can identify individuals at risk of complications and allow early intervention [15]. COVID-19-related conditions, such as post-acute sequelae of COVID-19 (PASC) [16], require appropriate attention and need to be managed primarily by generalists [17, 18]. In addition, general practitioners (GPs), as people’s confidants, have an important role to play in educating people about infection, prevention, and immunisation strategy [6, 11, 14]. In addition, maintaining good usual care can protect chronically ill people from deterioration through neglect at a time of public health emergency for the general population [6, 11, 14, 19].

In this way, pandemic-related collateral health risks can be mitigated [20]. The negative consequences of neglecting the core functions of primary care during a pandemic are examined in a Flemish study [21].

Several other authors propose an active role for GPs in developing plans for health emergency risks [7, 12, 13, 22, 23].

The role of Austrian GPs, as outlined in the 2022 update of the Austrian pandemic report [5], now includes primary and secondary prevention, treatment and management, rehabilitation, and public health tasks such as testing and vaccination. The need to maintain usual care for all patients in chronic and acute care is explicitly described.

In the pandemic plan, the following tasks of extramural care during a future pandemic were specified: Prevention of nosocomial infections by protective measures at the point of care and during transport; identification, care and treatment of persons infected with SARS-CoV-2; maintenance of standard care, ensuring access to and treatment of the acutely ill; ensuring access to and treatment of the chronically ill; maintenance of preventive services as far as the situation permits; protection of the inpatient sector against overload.

It should be emphasised that these tasks were empirically assigned to primary care based on experience during the public health emergency - that is, based on what GPs appeared to be doing or not doing at the time. There was no analysis of what had helped or hindered the fulfilment of the internationally agreed obligations of primary care as described above, partly due to the fact that there is no data generated by primary care in Austria, making it difficult to assess its achievements, shortcomings or needs.

No effort had been made to assess the availability of the resources needed to fulfil the various tasks, such as access to protective equipment, financial and structural support and human resources [11], as well as communication and cooperation channels between GPs, specialists and, for example, health authorities [24].

Against this background, the aim of this study was to provide a qualitative analysis of what helped or hindered the achievement of international goals for primary care in pandemic management in Austria.

## Methods

We conducted a qualitative study [25] using semi-structured interviews with general practitioners in different primary care settings (single practices, group practices, primary care centres) in Austria. A total of 30 semi-structured interviews were conducted between 2022.02.01 and 2022.07.22.

At the time we conducted the survey, doctors had been facing the challenges of COVID-19 for almost two years. At that time, the government regulations for COVID-19 were still in place, and three hard lockdowns had been implemented in 2020. The aim of the study was to understand the process of dealing with this situation. Therefore, the interviewees were first asked to think back and talk about the first phase of the pandemic and then asked how they assessed the situation at the time of the interview. This allowed us to gain an insight into their initial response and the process of refining the solutions chosen and adapting to the challenges. The open and qualitative approach of the interviews allowed the different challenges of the initial and subsequent response to the pandemic to be discussed, and doctors were free to elaborate on what they considered to be the greatest challenges in each area, without having to summarise too much.

### Sampling strategy

Following the extensive literature review that was conducted, the criterion of organisational setting was identified as highly influential in this field. Therefore, we chose a purposive sampling approach based on the criterion of organisational setting. We aimed to conduct the study with a heterogeneous sample of physicians working in single handed surgeries, group practices, and PHU (primary care unit) settings (see Table 1 for details) [26].

Physicians were recruited through the Austrian Society of General Practice (Österreichische Gesellschaft für Allgemein- und Familienmedizin, ÖGAM) via e-mail information and newsletters and through the research network of the Department of Health Services Research in Primary Care at the Medical University of Vienna. Of the 1350 physicians approached, 34 expressed their interest by email. They were contacted by telephone or e-mail. After a description of the topic and an introduction by the research team, their consent to participate in a qualitative interview was requested. Participants were sent a consent form and a short demographic questionnaire. Of these, 4 were lost to follow-up due to time constraints, and 30 returned the consent form and questionnaire. An interview date was then arranged. The doctors who responded were evenly distributed across the organisational setting dimension. The recruitment phase was therefore complete.

### Data collection

Interviews were conducted face-to-face, by telephone or via WebEx. The interviews were then recorded using an attached smartphone or the WebEx tool. The duration of the interviews ranged from 26 min to 1 h 25 min. The average duration was 56.5 min. None of the participants dropped out during the interview. It was always a one-to-one interview. No interview was interrupted or had to be repeated. The interviews were conducted by three interviewers, two of the co-authors (MM, NS) and CT. CT was a final year medical student and is mentioned in the acknowledgements. The interview guide was based on the six research questions in the supplementary material (S2). However, the order of the questions was modified after initial experience of the interviews. The 30 interviews were transcribed verbatim using Tucan software (contract, data protection agreement and data security agreement were concluded).

### Research questions and interview guide

The overarching research interest was how GPs in Austria were coping with the pandemic, firstly because GPs working on the front line are usually the first to be confronted with new challenges, and secondly because WHO has identified them as an important pillar of good pandemic management. Therefore, we also wanted to find out whether this pillar function was adequately supported in Austria.The research questions for this paper relate to two research topics from the interview guide, because we considered them to be the most challenging tasks during an airborne pandemic. The other topics from the questionnaire will be explored in separate publications.

- Infection control: protective equipment, practice organisation (appointment management, waiting room management), practice infrastructure, recommendations.
- Communication with authorities, health authorities, medical associations, colleagues, insurance companies, etc.

The interview guide was developed in conjunction with the WHO report on primary care and pandemic management “Role of primary care in the COVID-19 response” [6].

It was developed through a qualitative iterative process. First, key questions were derived directly from the research questions and literature review. In initial interviews, these questions were used in a very open-ended way, and enquiries arising from these interviews were noted. In a second step, the interview guide was refined and edited. Definite and potential follow-up questions were added in order to increase the comparability of further interviews. Thus, the guideline was developed from a very open to a semi-structured interview guideline [27].

### Data analysis

Given the highly exploratory nature of this study design, we aimed to achieve three objectives with our qualitative content analysis of the material. First, to analyse the interviews using pre-existing categories derived from the research questions, which in turn were based on existing research and theoretical background. Second, to uncover further knowledge from the interviews and use it for inductive category development [28]. Third, to explore differences in the material based on our sampling criterion of organisational form.

The analysis was primarily conducted by a research assistant (MM), with additional staff (KH, NS) independently coding approximately 30% of the interviews to ensure reliable and repeatable analysis of the material.

The coding was discussed and the codebook was developed in Excel with code name, description and parent category. Relevant quotations from the material were cited.

### Ethical aspects and data protection

The research team guarantees that the project was conducted in accordance with the Declaration of Helsinki (1964) and all subsequent updates of the Declaration. The team is responsible for ensuring that the project is conducted in accordance with the European Commission’s “Guidelines of Good Clinical Practice”, national requirements and with the requirements of the Medical University of Vienna and the Karl Landsteiner University of Health Sciences in Krems.

A positive vote for the survey from the Ethics Committee of the Med. University is available: EC no.: 1491/2021.

For all interviews, a written informed consent form as well as a written agreement to maintain anonymity and data protection were signed by the participants after they were informed in detail about the study.

## Results

### Description of interviewees

The interview sample consists of 30 participants. Participants were recruited from eight of the nine Austrian provinces. Participants could be recruited from all provinces except Salzburg (Table 1). The gender distribution was well balanced. Details are given in Table 1.

**Table 1:** Demographics of the participants

Variable	Subvariable	n
All		30
County	Burgenland	3
	Carinthia	1
	Lower Austria	4
	Upper Austria	2
	Salzburg	0
	Styria	4
	Tyrol	1
	Vienna	13
	Vorarlberg	2
Sex	Female	15
	Male	15
Type of practice	Single-handed (1 GP)	11
	Group-practice (2 + GPs)	11
	PVE (Primärversorgungseinheit,i.e. primary care unit)	8

### Results of the content analysis

Deductive and inductive analysis of the transcripts resulted in two main categories, each with between 3 and 5 subcategories. The corresponding main categories and sub-categories are shown in Table 2.

**Table 2:** Main categories and subcategories

Main categories	Subcategories
Practice management/care process adjustments	Practice organisation process adaptations in private practice
	Appointment management
	Infrastructure management
	Transmission control
	Personal protective equipment (PPE)
Communication management	Communication with institutions and authorities
	Information from ÖGAM
	Communication with colleagues

### Process adjustments in practice organisation in the private practice sector

None of the respondents closed their practices completely during the Corona pandemic. One doctor reported hostility from other doctors who closed their practices, and larger units reported taking on patients from closed practices in the area.

A sharp decline in patient attendance was noted by almost all participants. This was mainly attributed to patients’ fear of infection in the surgery.

The surgeries were empty and the patients stayed at home in shock (Interview 4).

20% drop in turnover (Interview 9).

Subsequently, however, an increase in patient contacts was reported. Process changes were also made in many organisations during the first days of the lockdown. These mainly involved separating patients spatially or temporally, adapting infrastructure and introducing protective equipment.

Of course, all this can only be done if the physical and human resources are available (Interview 1).

### Time management

In terms of time management, there was a strong trend towards working by appointment, especially when the patient had an infection (initially fewer patients, then many more). However, many practices interviewed did not change their appointment system in the context of the pandemic, as some had an appointment system in place before the pandemic and others had mixed systems. Among the mixed systems, different types were identified: patients with symptoms of infection were asked to make an appointment, while other patients could turn up without prior notice; an appointment system for all patients combined with free access for acute or infection-related problems; separate areas for patients with acute or other reasons for visiting; appointments for scheduled tests or therapies. Almost half of the 30 practices surveyed had switched to an appointment system because of Covid-19, with the intention of preventing transmission within their premises, with some of them setting up a fixed time slot for patients with signs of infection.

…through the appointment system to separate patients who are already sick or suspected of being sick from patients without symptoms of infection, because they need something else. And that is the first selection, which is done by telephone. Then they get their own appointment in the infection unit (Interview 15).

Only one doctor still does not offer appointments.

The following advantages were mentioned: less congestion in the waiting room; telephone contacts for appointments can already be used as a selection tool to assign specific appointments to patients with symptoms of infection, as well as to determine the urgency of the request.

Lack of acceptance by patients was mentioned as a disadvantage of the appointment system; one rural doctor also described meeting in the waiting room as a “mode of communication” that was missing because of the appointment system. Other disadvantages mentioned were that patients expect shorter waiting times for appointments and that there is more administrative work involved in arranging appointments.

### Infrastructure management

In many practices, access was restricted by physical barriers: first contact through a window; separate entrances to different areas; separate areas to isolate patients with infectious diseases.

Common measures to maintain physical distance included removing seating in waiting areas and spending time waiting outside the surgery.

We are in the middle of the city, patients have been informed, they can go for a walk for half an hour (Interview 2).

Several practices had the opportunity to create several waiting areas or treatment zones.

We were lucky to have this space, this infection room, so to speak (interview 8).

Problems mentioned were e.g. a single entrance to the premises, or small rooms.

What we would really like is more space, more rooms, more ways to separate patients (interview 26).

Coping strategies developed were: acquiring additional space (mostly provided by municipalities), contacting patients in their cars, practising in garages and tents:

We literally worked in parking lots, cars, garages, and separately set up party tents (Interview 19).

Physical contact with patients was reduced in many practices at the start of the pandemic, partly replaced by remote consultations by telephone or channelling through appointments.

### Control of transmission

One practice simply provided disinfectant dispensers, while others purchased sophisticated equipment or set up extra rooms. In some cases, the communication infrastructure was upgraded as the number of remote contacts (telephone or video) increased:

Very important are the communication structures, so the internet connection has to be very good, the telephone system has to be very well dimensioned, redundant (Interview 18).

Some doctors purchased additional mobile phones or laptops. Some practices set up home offices for some of their staff, especially at the beginning:

We also let our assistants work from home. Sometimes a doctor on rotation would also work from home, reading reports and things like that, so we could just unbundle the whole thing (Interview 18).

The most frequently mentioned infrastructure adaptation was the installation of transparent screens at reception desks. As a result, communication problems were reported, with a marked increase in noise levels. The installation of additional disinfectant dispensers was reported very frequently. Some practices were equipped with air filters or means of taking body temperature. Procedural changes to reduce transmission within the premises included regular airing, surface disinfection, more frequent changes of clothing and avoidance of physical contact, i.e. shaking hands was discouraged.

### Personal protective equipment (PPE)

Doctors reported a high level of uncertainty during the first phase of the pandemic.

How often do you have to change the mask? How infectious is it? And you were just very, very uncertain. And that was not pleasant in the beginning, yeah, you had to go through that (interview 21).

Concerns were expressed about the danger posed by the disease and the measures taken by the authorities:

It was clear that if we had a positive case of COVID-19 in the practice, the health authorities would immediately close the entire premises (interview 1).

Shortages of personal protective equipment at the start of the pandemic, especially disinfectants and masks, but also medical gloves, were overcome with makeshift solutions such as making their own disinfectants, reusing plastic raincoats, chemical suits and masks intended for different purposes. Masks were cleaned and reused.

Wearing masks was accepted by most as an important means of protection, with some mentioning communication barriers associated with this. Among other PPE, protective coats and gloves were well accepted. Full-body protection was used in several practices, most commonly in contact with suspected or known infectious patients and during the early pandemic period.

Other measures in practices were reported: testing of staff, information on the use of the FFP2 mask, disinfection, and keeping distance (notices; announcement in the community newspaper):

Of course we have informed the patients, also via our homepage, that they have to come to our practice equipped accordingly (interview 22).

### Communication management

Communication with institutions and authorities.

The first few weeks of the pandemic were perceived by all study participants to be particularly difficult in terms of information and guidance, although views differed on the later periods.

Communication between the different actors in the health sector was seen as a major challenge. One doctor expresses the sentiment described by a number of respondents:

I had the feeling that there was a fundamental interest from all sides and from all parties involved, whether it was the authorities, whether it was the colleagues, whether it was the medical association, to manage this situation well and jointly and positively (interview 1).

Nevertheless, there was some criticism of the communication structures, and information management was seen as unsatisfactory, particularly in the first few weeks.

In the first four to six weeks we didn’t hear from anybody. We just had what we had. (interview 15).

Complaints related to the lack of a contingency plan, recommendations that were inappropriate for extramural care, and inappropriate or incorrect recommendations (valve masks, practice closures). Understanding was expressed for the difficulties associated with the pandemic situation and the lack of knowledge about the virus and the disease:

It all took a bit of time, it’s chaos when a pandemic breaks out. [Who should I criticise now? No structure has been created for this (interview 27).

Overall, one doctor described a general problem in the cascade of communication in the context of this crisis, in which excessive demands and problems also occurred and were passed on at the institutional level.

One level simply tries to pass on unresolved problems to another level, then of course it becomes problematic. […] If a structure is overburdened, then exactly these communication problems occur (interview 1).

For most doctors, the Austrian Medical Association was the main point of contact. The Austrian Medical Association represents all Austrian doctors and membership is compulsory. Half of the respondents saw efforts to maintain a certain flow of information, e.g. through newsletters, video conferences and advice. However, some respondents indicated that they only had positive experiences because they had personal contacts in the medical association.

The other half report negative experiences with their representatives. Respondents say that individual questions were not answered or were answered slowly and not always correctly. They felt left alone and not well involved.

Another important stakeholder is the Austrian Health Insurance Fund (ÖGK), which is the contracting authority for all physicians in the public health system in extramural care. Several doctors said they had good contact with the ÖGK, while others said they had little, or only for operational matters such as billing and administration. Others, however, feel left alone:

The ÖGK, ……, was and is switched off as far as the pandemic is concerned. No guidelines, no instructions, nothing. So nothing, in two years nothing. The information you get is where you can bill (interview 14).

Another important stakeholder mentioned was the health authorities at regional level. Again, the spectrum was quite wide: some interviewees described good contact - some of them suspected that this was due to good personal connections. Others reported a generally slow and untimely response to COVID-19, as well as poor accessibility of these institutions and erroneous or outright incorrect messages:

We took it for granted that the authority is the authority and we are at the mercy of the authority. It sounds bad, but it is true. If you call two different health authorities and really get statements that contradict each other - in Vienna - then I can’t argue with that anymore (interview 7).

### Communication between doctors

The potential for self-help within the profession was mentioned as a positive aspect, as was the gathering and sharing of information and procedures and techniques for coping with the challenge, albeit with the disadvantage of very individual, non-systematic solutions.

We are doing well, but I think it is still far from quality management, because everyone does what they want (interview 26).

All interviewees stated that communication within their own offices was very important and was perceived as positive in almost all practices.

I realise more and more that being a doctor is very, very important, so one of the central tasks is how you work with the team and that you create an environment where they can work. Where communication can flow (interview 9).

Collaboration within a team was identified as an important factor in resilience to the COVID crisis.

So for me it was actually not the worst time of my life, but it also showed how strong we are as a team and what we can achieve (interview 29)

Colleagues were mentioned as the main interlocutors. For example, most interviewees spoke of good networking between doctors and helpful contacts via communication platforms such as WhatsApp and/or Facebook groups.

There was a huge exchange of knowledge among us colleagues or GPs, which we probably never thought would happen (interview 15).

Some doctors expressed a wish for better networking and criticised a lack of structured information sharing. Communication with specialists was seen as positive, as was communication with hospitals and nursing homes. Even though continuing medical education (CME) could be completed online, the interviewees missed meeting each other in face-to-face events.

### Information from the Austrian society for general and family medicine (ÖGAM)

The information provided by the Austrian Society for General Practice and Family Medicine (ÖGAM) through its networks, such as newsletters, podcasts and online information platforms, was considered very supportive. The ÖGAM is made up of voluntary members and has about a third of Austrian GPs on its list.

We always received very good, very good materials from the Austrian Society of General Medicine. I really appreciated that (Interview 21).

Some more informal sources of information were mentioned by the participants. One person each mentioned the Tyrolean Society for General Medicine (member association of the ÖGAM), the Primary Care Forum and the medical universities as sources of supportive information. All of them communicate through their informal networks.

## Discussion

The responses we collected show a wide range of creative, individual solutions to the problems GPs faced in the early stages of the pandemic. Despite official warnings, Austrian GPs had not closed their practices even at the start of the pandemic, unless they had been shut down by the authorities for quarantine. This perception of our interviewees is confirmed by figures from the health insurance funds, which show only a slight reduction in the number of GP practices opened compared with the years before the pandemic, as reported by the Austrian Court of Audit 21, the official auditor of the public sector. GPs have adapted well to the challenge, showing a high degree of flexibility and creativity and proved able to maintain access to health care. This observation has been made in several other studies in different countries [7, 8, 12, 13], regardless of the health care system.

Respondents to our survey observed that patients were reluctant to seek consultations at the beginning of the pandemic, whereas access to the health system at primary care level returned to almost normal in the later stages. This was confirmed for Germany [29], a country with a similar health system, as well as by data from the Austrian health insurance system. These data show some decline at the beginning of the first closure and a milder one during the second closure [30]. After this initial, brief drop in the number of patients, there was a significant increase in patient consultations. The initial drop was felt by the participating GPs to be due not only to the lockdowns, but also to patients’ fears. This is also in line with the findings in other studies [11]. Part of the GPs` efforts were therefore devoted to the maintenance of usual care for people with acute health problems not related to COVID-19, and for the chronically ill. Austrian primary care practices, like many in the industrialised countries, increased their use of virtual consultations by telephone or video [11, 29] or made home visits [12] to those who did not feel safe enough to physically visit a practice. The fact that the decline in consultations and the number of surgeries closed was significantly higher among specialists in all early phases of the pandemic [5] can be discussed as a reflection of their mostly long-standing doctor-patient relationship, and a high intrinsic motivation of general practitioners - even though they are not legally obliged in Austria to ensure the maintenance of patient care.

Austria is one of the few countries in the EU that does not derive health data from medical records in primary care. As a result, it was not only on the doctors’ side that there was an astonishing lack of information about which and how many primary (or secondary) care surgeries had actually been opened: According to the report of the Austrian Court of Audit, neither the Ministry of Health nor the regional authorities were able to find out whether and how the outpatient care structures were functioning. This may also have been one of the reasons why, for a considerable period of time, extramural health care staff did for a rather long period not receive the necessary amount of personal protective equipment [30]. Yet also in countries with strong primary care, physicians reported considerable difficulties in cooperating with the authorities [9]. Primary care not being part of a pandemic plan does not seem to be limited to countries with weak primary care systems [11, 13]. Kraus et al. [12], comparing five different countries with different health care systems, found that primary care was apparently rather consistently no priority in policy making, and concluded that the strengths of primary care were generally underutilised in the management of the pandemic situation. However, strong primary care systems were found to have had some advantage, providing financial and structural support [8] .

Another prominent issue was information transfer, which was identified by several studies besides ours as one of the central difficulties. Our survey suggests that information provided by a special, primary care oriented Austrian platform and newsletters for primary care [31] can be helpful. This is supported by a Norwegian paper that shows the importance of information coming from primary care experts. Still only a minority of Austrian GPs could be reached through these channels. The difficulties in disseminating of these tools can partly be explained by the fact that there is no communication structure that allows to contact all GP practices directly and simultaneously. The Austrian Medical Association (ÖÄK) must use 9 different communication networks in 9 different federal states, the Ministry of Health has no direct access to physicians at all.

### Strengths and limitations of the study

#### Strengths

This study is the first of its kind in Austria and provides a picture of the situation in Austrian primary care that cannot and has not been obtained elsewhere. Our results made it possible to compare Austrian performance with that of other European health care systems. The study provides information about the efforts of Austrian GPs to work without formalising their role, and about the obstacles and barriers they encounter. We were able to conduct 30 semi-structured interviews on a wide range of issues of concern to this group during a major health care crisis. We used a structured interview guide and clearly reached data saturation. We collected qualitative data from interviewees until enough data had been collected to provide sufficient insight into the topic to answer the research question. Collecting more data would not have added value.

#### Limitations

As a qualitative study, it can only provide a first insight into the situation. Recall and memory bias must be taken into account, particularly as the period studied was one of high workload, rapid turnover of tasks and information, and considerable confusion, all of which may have influenced and ultimately distorted participants’ views.

The recruitment strategy via the ÖGAM mailing list created a high threshold. In fact, the response rate was low. We cannot say how many GPs were reached by the emails, how many opened them or simply clicked away, or how many were not interested in the topic. The need to actively apply to be interviewed will also have been a barrier. All this might have resulted in the participation of doctors with a high level of interest in the topic and intrinsic motivation - perhaps even those with smaller practices, less workload, and more time.

This may have led to an overestimation of resilience and coping skills. We tried to correct for this by looking at the little data available on the situation and were able to confirm some of the participants’ perceptions. This may increase the reliability of our findings.

## Conclusion

This study provides a first insight into the experiences of GPs in Austria, a country with a weak primary care system, and their functioning within a health care system that has so far refrained from defining a primary care level and its responsibilities.

The conclusion of our qualitative analysis is that GPs in all organisational forms in Austria showed great ingenuity and flexibility to maintain the care of their patients and to provide additional protection against infection.

The pandemic was characterised by a high degree of uncertainty in all areas. For GPs, there was a sense of responsibility from the outset. GPs knew that their presence was needed to care for patients with and without COVID-19, to care for the chronically ill, to prevent infection in their practices, and to care for the community. They were well aware of the tasks of primary health care, even if these were not defined for Austrian general practitioners before or during the pandemic.

All our findings are very much in line with other studies. GPs in all the European countries surveyed seem to have shown great resilience under adverse conditions, finding creative, innovative, and effective solutions. Primary care-based systems seem to have had advantages over unstructured systems such as the Austrian one. These lessons should lead to a move towards more structured care and a stronger role for primary care. A pandemic plan must explicitly describe the role of primary care to ensure that this part of the health system is not under-utilised. Such a plan should draw on the expertise of primary care providers, who can and have made a significant contribution to managing the pandemic situation. This is a valid conclusion of our survey not only for Austria, but also for other countries.

### Electronic supplementary material

Below is the link to the electronic supplementary material.

**Supplementary Material 1:** COREQ Checklist

**Supplementary Material 2:** Interview Guide

## Data Availability

All data are centrally stored on the server of the Medical University of Vienna. All data was anonymized before the analysis. Reasonable request to corresponding author is required to access non-identifiable data by external users Access will be subject to a data transfer agreement.

## List of abbreviations

GP: General Practitioner
PHC: Primary Health Care
PC: Primary Care
ÖÄK: Österreichische Ärztekammer (Austrian Physicians` Association)
ÖGAM: Österreichische Gesellschaft für Allgemein- und Familienmedizin (Austrian Society of General Medicine and Family Practice)
ÖGK: Österreichische Gesundheitskasse (Austrian Health Insurance Fund)
