# Supplementary material for: “We literally worked in parking lots, cars, garages, and separately set up party tents”: qualitative study on the experiences of GPs in the frame of the SARS-CoV-2 pandemic in Austria
Source: BMC Health Serv Res. 2023 Dec 12;23:1394. doi: 10.1186/s12913-023-10363-4 (PMC10714610; doi:10.1186/s12913-023-10363-4)
Supplement: Supplementary file 1 — Supplementary Material 1: COREQ Checklist [file 12913_2023_10363_MOESM1_ESM.docx]

**Manuscript:**

**“We literally worked in parking lots, cars, garages, and separately set up party tents.” Qualitative study on the experiences of GPs in the frame of the SARS-CoV-2 pandemic in Austria**

**Consolidated criteria for reporting qualitative studies (COREQ): 32-item checklist**

Developed from:

Tong A, Sainsbury P, Craig J. Consolidated criteria for reporting qualitative research (COREQ): a 32-item checklist for interviews and focus groups. *International Journal for Quality in Health Care*. 2007. Volume 19, Number 6: pp. 349 – 357

| **No. Item** | **Guide questions/description** | **Reported on** |
| --- | --- | --- |
| **Domain 1: Research team and reﬂexivity** |  |  |
| *Personal Characteristics* |  |  |
| 1. Interviewer/facilitator | Which author/s conducted the interview or focus group? | Method section, data collection |
| 2. Credentials | What were the researcher’s credentials? E.g. PhD, MD | Method section, data collection and Author’s Information section |
| 3. Occupation | What was their occupation at the time of the study? | Method section, data collection and Author’s Information section |
| 4. Gender | Was the researcher male or female? | Method section, data collection |
| 5. Experience and training | What experience or training did the researcher have? | Method section, data collection and Author’s Information section |
| *Relationship with participants* |  |  |
| 6. Relationship established | Was a relationship established prior to study commencement? | Method section, sampling strategy |
| 7. Participant knowledge of the interviewer | What did the participants know about the researcher? e.g. personal goals, reasons for doing the research | Method section, sampling strategy |
| 8. Interviewer characteristics | What characteristics were reported about the interviewer/facilitator? e.g. Bias, assumptions, reasons and interests in the research topic | Method section, sampling strategy |
| **Domain 2: study design** |  |  |
| *Theoretical framework* |  |  |
| 9. Methodological orientation and Theory | What methodological orientation was stated to underpin the study? e.g. grounded theory, discourse analysis, ethnography, phenomenology, content analysis | Methods section overall and data analysis |
| *Participant selection* |  |  |
| 10. Sampling | How were participants selected? e.g. purposive, convenience, consecutive, snowball | Method section, sampling strategy |
| 11. Method of approach | How were participants approached? e.g. face-to-face, telephone, mail, email | Method section, sampling strategy |
| 12. Sample size | How many participants were in the study? | Method section, sampling strategy |
| 13. Non-participation | How many people refused to participate or dropped out? Reasons? | Method section, sampling strategy |
| *Setting* |  |  |
| 14. Setting of data collection | Where was the data collected? e.g. home, clinic, workplace | Method section, data collection |
| 15. Presence of non-participants | Was anyone else present besides the participants and researchers? | Method section, data collection |
| 16. Description of sample | What are the important characteristics of the sample? e.g. demographic data, date | Result section, Table 1 |
| *Data collection* |  |  |
| 17. Interview guide | Were questions, prompts, guides provided by the authors? Was it pilot tested? | Supplementary material 2 (S2) |
| 18. Repeat interviews | Were repeat interviews carried out? If yes, how many? | Method section, data collection |
| 19. Audio/visual recording | Did the research use audio or visual recording to collect the data? | Method section, data collection |
| 20. Field notes | Were ﬁeld notes made during and/or after the interview or focus group? | No, no field notes were made. |
| 21. Duration | What was the duration of the inter views or focus group? | Method section, data collection |
| 22. Data saturation | Was data saturation discussed? | Discussion section, strength and limitations |
| 23. Transcripts returned | Were transcripts returned to participants for comment and/or correction? | No. |
| **Domain 3: analysis and ﬁndings** |  |  |
| *Data analysis* |  |  |
| 24. Number of data coders | How many data coders coded the data? | Methods section, data analysis |
| 25. Description of the coding tree | Did authors provide a description of the coding tree? | Findings, table 2 |
| 26. Derivation of themes | Were themes identiﬁed in advance or derived from the data? | Methods section, data analysis: both we coded inductively and deductively |
| 27. Software | What software, if applicable, was used to manage the data? | Methods section, data collection & data analysis |
| 28. Participant checking | Did participants provide feedback on the ﬁndings? | No. |
| *Reporting* |  |  |
| 29. Quotations presented | Were participant quotations presented to illustrate the themes/ﬁndings? Was each quotation identiﬁed? e.g. participant number | Result section |
| 30. Data and ﬁndings consistent | Was there consistency between the data presented and the ﬁndings? | Result section |
| 31. Clarity of major themes | Were major themes clearly presented in the ﬁndings? | Result section |
| 32. Clarity of minor themes | Is there a description of diverse cases or discussion of minor themes? | Result section |
