# Supplementary material for: “We literally worked in parking lots, cars, garages, and separately set up party tents”: qualitative study on the experiences of GPs in the frame of the SARS-CoV-2 pandemic in Austria
Source: BMC Health Serv Res. 2023 Dec 12;23:1394. doi: 10.1186/s12913-023-10363-4 (PMC10714610; doi:10.1186/s12913-023-10363-4)
Supplement: Supplementary file 2 — Supplementary Material 2: Interview Guide [file 12913_2023_10363_MOESM2_ESM.docx]

##

**Interviewguide Cov-FIT study**

How did you and the ordination fare at the beginning and during the course of the COVID-19 pandemic with regard to ...?

1. Infection control: protective equipment, practice organization, practice infrastructure

- Experiences, hurdles and opportunities, lessons learned, where would support be needed, where was support provided?

2. Remote consultations

- Experiences, hurdles and opportunities, lessons learned, where would support be needed, where was there support? (technical, organizational, financial, content).

3. Diagnostics, testing, reporting, sick leave, monitoring and treatment of COVID-19 patients

- Experiences, hurdles and opportunities, lessons learned, where would support be needed, where was there support?

4. Treatment of patients with chronic and other diseases

- Experiences, hurdles and opportunities, lessons learned, where would need support, where was there support?

5. Covid-Vaccination

- Experiences, hurdles and opportunities, lessons learned, where would need support, where was there support?

6. Communication: with authorities, colleagues etc.

- Experiences, hurdles and opportunities, lessons learned, where would support be needed, where was there support?
